# Frequency of seizures in post-cerebrovascular accident patients

**DOI:** 10.12669/pjms.42.9.16727

**Published:** 2026-09

**Authors:** Mirza Raees, Sheraz Saleem, Muhammad Adnan, Areeba Mariam Mehmood

**Affiliations:** 1 Dr. Mirza Raees, MBBS, FCPS Trainee, Department of Medicine, Unit 1, Faisal Masood Teaching Hospital, Sargodha, Punjab, Pakistan; 2 Dr. Sheraz Saleem, MBBS, FCPS, Department of Medicine, Unit 1, Faisal Masood Teaching Hospital, Sargodha, Punjab, Pakistan; 3 Dr. Muhammad Adnan, MBBS, M.D Trainee, Department of Medicine, Unit 1, Faisal Masood Teaching Hospital, Sargodha, Punjab, Pakistan; 4 Dr. Areeba Mariam Mehmood, MBBS, Department of Medicine, Unit 1, Faisal Masood Teaching Hospital, Sargodha, Punjab, Pakistan

**Keywords:** Brain Ischemia, Cerebrovascular Disorders, Epilepsy, Cerebral Hemorrhage, Hemorrhagic Stroke, Ischemic Stroke, Risk Factors, Seizures, Stroke

## Abstract

**Objectives:**

To determine the frequency of seizures in patients following a cerebrovascular accident (CVA).

**Methodology:**

This prospective observational cohort study was carried out at the Department of Medicine, Faisal Masood Teaching Hospital, Sargodha, between July 2024 and April 2025. After ethical clearance and written consent, 73 adults of either gender, aged 20 to 70 years, who reached the hospital within 24 hours of an acute stroke were enrolled. Data were analyzed using SPSS version 26.0.

**Results:**

Mean age was 55.33 ± 14.32 years, with 43 men (58.9%) and 30 women (41.1%). Ischemic stroke was seen in 49 patients (67.12%) and hemorrhagic stroke in 24 (32.88%). The middle cerebral artery was the most common territory, with 30 cases (41.1%), followed by the anterior cerebral artery with 24 (32.88%), the posterior cerebral artery with 10 (13.7%), and the cortical area with 9 (12.33%). Post-stroke seizures developed in 40 patients (54.79%), with onset ranging from 2 to 49 days (mean 28.03 ± 14.66). Late-onset seizures occurred in 30 patients (41.1%) and early-onset seizures in 10 (13.7%). On binary logistic regression, anterior cerebral artery involvement was an independent predictor of seizures (OR 4.88, 95% CI 1.43–18.67, p = 0.014); all patients with posterior cerebral artery or cortical involvement developed seizures (complete separation, odds ratios not estimable), whereas age, gender, and stroke type were not significant (model AUC 0.84).

**Conclusion:**

Seizures developed in over half of the stroke patients, and a late-onset pattern was more common. Cortical and posterior cerebral artery involvement showed the strongest association with seizures, and anterior cerebral artery involvement was an independent predictor of seizure occurrence.

## INTRODUCTION

Post-stroke seizures (PSS) are among the most frequent neurological complications seen after a cerebrovascular accident and are a major cause of disability and poor recovery. The chances of developing seizures increase several times after a stroke, especially when the cortical part of the brain is involved. Stroke severity, location, and type strongly influence seizure risk.^1^,^2^ Early-onset seizures typically occur within two weeks of the stroke, while late-onset seizures develop after this period. Both types may negatively influence rehabilitation and long-term functional outcomes.^3^,^4^

The pathophysiology of PSS involves brain irritation and tissue damage due to decreased blood supply. The lack of oxygen in neurons renders them electrically unstable, producing abnormal electrical discharges. Early-onset seizures are mostly associated with acute injury, swelling, and metabolic disturbances, whereas late-onset seizures are associated with scarring and long-term alteration of brain tissue.^5^-^7^ Patients with late-onset seizures have a higher risk of developing post-stroke epilepsy and require long-term follow-up and treatment. Some studies indicate that post-stroke seizure patients might not recover their cognitive ability as quickly and are more likely to develop memory issues or dementia in later life.^8^,^9^

A study by Ferreira-Atuesta et al. followed 1,225 patients who had suffered a stroke and found that 5% developed acute symptomatic seizures. In contrast, 6% developed post-stroke epilepsy over a median follow-up of 1.6 years. Seizure occurrence was linked to the severity, cause, and location of the stroke. Early electroencephalogram (EEG) findings were not useful in predicting future epilepsy. Interestingly, patients who received reperfusion therapy did not show a higher risk of seizures.^10^ In contrast, a study by Siddiqi et al. found a much higher occurrence of early-onset seizures in 58% of their patients, with generalised seizures seen in 74% and multiple seizure episodes in 62% of cases. A statistically significant relation (p < 0.05) was found between late-onset seizures and the presence of ischemic heart disease, a prior stroke, or high blood pressure. These results indicate that the vascular risk factors can be a powerful stimulus for the late-onset seizures and post-stroke epilepsy.^11^ International statistics indicate that seizure rates are lower. In contrast, regional investigations tend to report higher rates because of delayed stroke presentation, limited EEG availability, the shortage of rehabilitation services, and the increased prevalence of uncontrolled risk factors, including hypertension and diabetes.

Although post-stroke seizures are a common complication, the condition has not been well studied in developing countries. Local data are required to determine the prevalence of these seizures and the risk factors most applicable to the local population. The frequency, timing, and lesion-based risk factors of post-stroke seizures are not well defined in the Pakistani population, and this gap is the reason the present study was carried out. Early detection of such patients enables timely management before they deteriorate further neurologically. This study was conducted to determine the frequency of seizures in patients admitted with cerebrovascular accidents and to characterise their timing, type, and other clinical features. Knowledge of post-stroke seizure patterns can help enhance patient follow-up, facilitate early treatment, and improve the quality of life of stroke survivors.

## METHODOLOGY

This prospective observational cohort study was conducted in the Department of Medicine, Faisal Masood Teaching Hospital, Sargodha, between July 2024 and April 2025. Non-probability consecutive sampling was used to select 73 patients. The sample size was estimated using the formula for a single population proportion, n = Z²P(1−P)/d², with Z = 1.96 at a 95% confidence level, an expected seizure frequency (P) of 74% taken from a previous regional study^11^, and an absolute precision (d) of 10%.

### Inclusion criteria:

- Both male and female patients aged between 18 and 70 years who presented with an acute cerebrovascular accident (CVA) within the preceding 24 hours.

### Exclusion criteria:

- Patients with subarachnoid haemorrhage, subdural hematoma, arteriovenous malformations, inflammatory vascular disease, transient ischemic attacks, pseudostroke due to metabolic causes, brain tumours, migraines, or psychiatric illness.
- Patients with a previous history of epilepsy, prior anticonvulsant therapy, previous intracranial surgery, previous traumatic brain injury, or seizures related to drug abuse or alcohol withdrawal were also excluded.

A total of 98 patients were screened, of whom 25 were excluded for not meeting the eligibility criteria or declining participation. The remaining 73 eligible patients were recruited consecutively from the medical ward and emergency department. Participants lost to follow-up were replaced with consecutively recruited eligible patients, resulting in a final analyzed sample of 73 (Fig.1).

**Fig.1:**
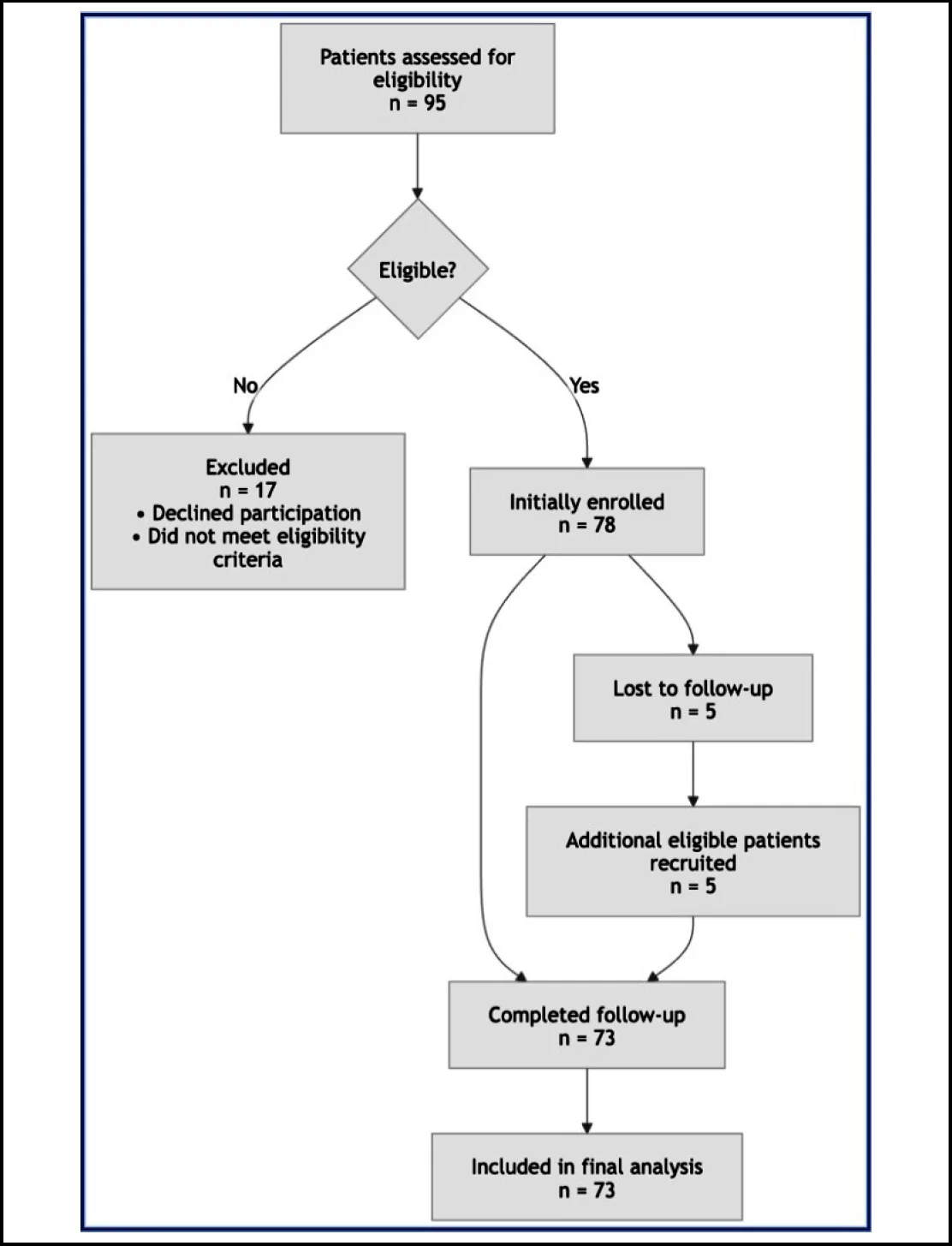
Flow diagram of the patients according to Strobe Guidelines

### Ethical Approval:

The hospital’s research committee granted ethical approval (Letter No. 1121/SMCTH/DHQ, dated: October 12, 2023). The study was conducted in accordance with the Declaration of Helsinki. All participants or their legal guardians were informed and gave informed consent before inclusion.

Demographic information was taken (name, age, gender, type of stroke, and the vessel involved (middle cerebral artery, anterior cerebral artery, posterior cerebral artery, or cortical region). The diagnosis of stroke was made according to the sudden neurological symptoms such as weakness, numbness, speech difficulties or loss of consciousness, which were confirmed by CT scans that revealed ischemic infarction or hemorrhage.^12^,^13^

All patients were followed up for three months following the stroke. A consultant physician with over five years of clinical experience monitored seizure activity during this period. Follow-up was conducted through scheduled outpatient visits at the end of the first and third months, supplemented by telephone contact; any patient who could not be reached was recorded as lost to follow-up. Symptoms that were used to identify post-stroke seizures, including muscle jerks, loss of awareness, altered sensations, or unusual movements, as per International League Against Epilepsy (ILAE) definitions, were confirmed by the consultant physician.^8^,^12^ An electroencephalogram was obtained only when the clinical diagnosis was uncertain. These seizures were categorized into two groups: early-onset, which happened within 14 days of stroke, and late-onset, which happened after 14 days. The seizure episodes were documented and treated in accordance with the hospital’s standard clinical practice.

Data were analyzed in SPSS 26.0 and R Studio. The chi-square test was used to compare seizures and clinical or demographic variables. A p-value of ≤0.05 was considered significant. Binary logistic regression was used to estimate adjusted odds ratios with 95% confidence intervals.

## RESULTS

The study enrolled 73 patients aged 20 to 70 years (mean 55.33 ± 14.32 years), of whom 43 (58.9%) were male and 30 (41.1%) female. Ischemic stroke was present in 49 patients (67.12%) and hemorrhagic stroke in 24 (32.88%). Middle cerebral artery involvement was most common (41.1%), followed by anterior cerebral artery (32.88%), posterior cerebral artery (13.7%), and cortical involvement (12.33%) (Table-I).

**Table-I:** Baseline Demographic and clinical characteristics of study patients (n=73).

Parameter	N	%
Age (Years), Mean ± SD	55.33 ± 14.32	—
Age Range (Min–Max)	20–70 years	—
***Age Groups***		
20–35 years	9	12.33%
36–50 years	14	19.18%
51–65 years	26	35.62%
>65 years	24	32.88%
***Gender***		
Male	43	58.90%
Female	30	41.10%
***Type of Stroke***		
Ischemic Stroke	49	67.12%
Hemorrhagic Stroke	24	32.88%
***Vessel Involvement***		
Middle Cerebral Artery	30	41.10%
Anterior Cerebral Artery	24	32.88%
Posterior Cerebral Artery	10	13.70%
Cortical Involvement	9	12.33%

Post-stroke seizures were observed in 40 (54.79%; 95% CI 43.4–65.7%) patients, while 33 (45.21%) did not develop any seizures during the follow-up period. Among those who developed seizures, the time of onset ranged between 2 and 49 days, with a mean of 28.03 ± 14.66 days. Early-onset seizures, occurring within 14 days of stroke, were noted in 10 patients (13.7%), while late-onset seizures, developing after 14 days, were recorded in 30 patients (41.1%). The details of seizure characteristics are presented in Table-II.

**Table-II:** Post-Stroke Seizure Characteristics among Affected Patients

Seizure Variable	N	%
***Post-Stroke Seizure***		
Yes (Seizure occurred)	40	54.79%
No (No seizure)	33	45.21%
***Seizure Type (among n=40)***		
Early-Onset (within 14 days)	10	13.70%
Late-Onset (after 14 days)	30	41.10%
***Onset Timing (days)***		
Mean ± SD	28.03 ± 14.66 days	—
Range (Min–Max)	2–49 days	—

**Table-III:** Binary logistic regression: predictors of post-stroke seizure (n = 73)

Predictor	Category	β (log-odds)	Adjusted OR	95% CI	p
Constant		−1.482	0.227	0.012–3.185	0.291
Vessel involvement	MCA (ref)	—	1	—	—
	Anterior cerebral artery	1.586	4.88	1.43–18.67	0.014
	Cortical involvement	19.81	*	*	0.993
	Posterior cerebral artery	19.82	*	*	0.992
Age (per year)		0.00227	1	0.96–1.05	0.914
Gender	Female (ref)	—	1	—	—
	Male	0.01917	1.02	0.30–3.54	0.976
Stroke type	Hemorrhagic (ref)	—	1	—	—
	Ischemic	0.1799	1.2	0.31–5.13	0.8

* Cortical and PCA odds ratios are not estimable by standard maximum likelihood because all patients in these groups had seizures (9/9 and 10/10), producing perfect separation.

In a binary logistic regression model in RStudio, vessel territory was the only independent predictor of post-stroke seizures. Relative to middle cerebral artery involvement, the adjusted odds of seizure were significantly higher with anterior cerebral artery involvement (OR 4.88, 95% CI 1.43–18.67, p = 0.014). Age (OR 1.00, 95% CI 0.96–1.05, p = 0.914), male gender (OR 1.02, 95% CI 0.30–3.54, p = 0.976), and ischemic stroke relative to hemorrhagic (OR 1.20, 95% CI 0.31–5.13, p = 0.800) were not significant. The model was significant overall (likelihood-ratio χ²(6) = 35.41, p < 0.001), with a Nagelkerke R² of 0.51 and an area under the ROC curve of 0.84.

## DISCUSSION

This study included 73 stroke patients and found post-stroke seizures in 40 (54.79%) of cases, with most patients being male [43 (58.9%)] and aged between 51 and 65 years. Ischemic stroke was more common, seen in 49 (67.12%) of patients, while 24 (32.88%) had hemorrhagic stroke. The middle cerebral artery was the most frequently involved vessel [30 (41.1%)], followed by the anterior, posterior, and cortical regions. Late-onset seizures were more frequent 30 (41.1%) than early-onset seizures 10 (13.7%), with the average time of onset being 28.03 ± 14.66 days. In logistic regression, age, gender, and stroke type were not independent predictors of seizure occurrence, whereas anterior cerebral artery involvement independently predicted seizures (OR 4.88, p = 0.014) and all patients with posterior cerebral artery or cortical involvement developed seizures, underscoring the role of lesion location. The regression model showed good discrimination (AUC = 0.84).

Ferreira-Atuesta et al. studied 1,225 patients and reported a reduction in seizures. Approximately 5% experienced acute symptomatic seizures, and 6% had post-stroke epilepsy after 1.6 years of follow-up. Their analysis associated the severity, cause, and location of stroke with seizures, but initial EEG measurements were not applicable in the prediction of epilepsy, and reperfusion therapy had no impact on the risk of seizures.^10^ Conversely, Siddiqi and co-researchers identified early seizure in 58 per cent of the patients, generalised seizure in 74 per cent, and multiple episodes in 62 per cent. Ischemic strokes were more common (80%), and late-onset seizures were attributed to high blood pressure, heart diseases, and prior stroke (p < 0.05).^11^ Current study results were more similar to those of Siddiqi since the current study also found more seizures in ischemic stroke patients and those with vascular risk factors, but the current study cases had fewer generalised seizures and a more significant association with cortical involvement. In contrast to the study by Ferreira-Atuesta, the current study’s data indicated that anatomical factors played a stronger role than the type of treatment in identifying seizure risk.^10^

Khan and colleagues reported a lower post-stroke seizure incidence of 8% in a series of 200 patients, with 3% of early and 5% of late-onset seizures. Most of the initial seizures were generalised tonic-clonic, and the later ones were partial; 1.5% of the patients had epilepsy. In the current study, the overall rate was higher, which may be explained by patient selection, stroke severity, or a shorter follow-up period. The two studies also concurred that early seizures are generalised, whereas late-onset seizures are partial and exhibit a similar trend across various populations.^14^

Lidetu et al. and Zewdu et al.^15^ studied 568 patient records and discovered the seizure episodes to be 22.18%. They stated that the important predictors were older age, hemorrhagic stroke, surgery, and the use of tramadol. Although age was also a factor in the current study, ischemic strokes and involvement of the cortical vessels were more strongly associated with post-stroke seizures. 15 Such differences may be attributed to variations in stroke subtype distribution and differing clinical practices across hospitals. Conrad and his colleagues examined 593 patients and discovered that 11.6% of them experienced seizures in 30 months of follow-up. Their research revealed that seizures were associated with younger age, high stroke scores, and clotting issues.^16^ They also reported that hemorrhagic strokes and sinus thrombosis had more frequent seizures. Unlike the current study, they did not identify stroke location (cortical vs. subcortical) as a significant predictor of seizures, a discrepancy that may reflect differences in imaging protocols or case selection. The present study consistently found cortical involvement to be strongly associated with post-stroke seizures, with all such patients developing seizures.

Agarwal et al. examined 291 patients with ischemic stroke and discovered that early seizures occurred in 12.7% of patients. Cortical stroke (OR = 4.2), large artery disease (OR = 2.9), high disability scores, anticoagulant use, and hypertension were all related to seizure risk.^17^ Those with early seizures had worse outcomes at three months. Their results support current study findings on cortical involvement and ischemic strokes, though the current study also included late-onset seizures. Shariff and co-researchers studied 594 patients and reported a 13.8% seizure rate. They found seizures linked to anterior circulation infarcts, cortical strokes, large artery atherosclerosis, ischemic heart disease, and brain surgery (p < 0.05).^18^ Stroke severity and loss of consciousness at presentation were also major predictors. The current study had a higher seizure rate but similar findings on cortical and ischemic stroke involvement. The current study also observed that all patients with posterior cerebral artery involvement developed seizures, adding a new factor not reported in their study.

The current study found a higher rate of total seizures with more late-onset cases. A review by Camilo et al. and Goldstein et al.^19^ showed that early seizures occurred in 2–33% of cases, while late seizures ranged from 3–67%, with post-stroke epilepsy seen in 2–4% of patients.^19^ Cortical involvement and stroke severity emerged as consistent risk factors across all studies reviewed, a pattern now firmly established by large-scale meta-analyses. Nandan et al. analyzed 128 studies and reported an overall incidence of 7% for early post-stroke seizures and 10% for post-stroke epilepsy, with cortical involvement carrying an odds ratio of 3.09 for early seizures and 3.20 for post-stroke epilepsy.^20^ The considerably higher seizure rate observed in the current cohort (54.79%) likely reflects the tertiary referral nature of the study centre, inclusion of consecutive symptomatic patients irrespective of severity, and the high burden of uncontrolled vascular risk factors prevalent in the local population. The short three months window also concentrated case detection, and the lack of routine outpatient seizure prophylaxis in this setting may have allowed more seizures to be observed than in cohorts from better-resourced systems. A recent retrospective study by Pozo Putalivo et al. in 760 stroke patients corroborated cortical involvement and early seizures as key independent predictors of post-stroke epilepsy; hemorrhagic stroke patients in that cohort carried a markedly higher cumulative seizure risk compared to ischemic patients, 37% by 10 months versus 10% by 20 months, respectively.^21^ Although the present study did not detect a statistically significant difference in seizure rate between the hemorrhagic and ischemic stroke groups, this may be attributable to the relatively small sample size and warrants confirmation in larger prospective studies.

The prognostic significance of post-stroke seizures extends well beyond the immediate post-ictal event. A meta-analysis of ten cohort studies comprising 177,929 stroke patients by Ren et al. demonstrated that post-stroke epilepsy is independently associated with a 68% higher risk of all-cause mortality (OR 1.68, 95% CI 1.51–1.87, p < 0.001), with this finding remaining consistent across both ischemic and hemorrhagic subtypes and irrespective of follow-up duration.^22^ These data reinforce the clinical imperative for systematic seizure risk stratification in stroke survivors. Validated prediction tools provide a practical framework, i.e., the SeLECT score, developed and validated for ischemic stroke, achieves an area under the curve (AUC) of 0.77, while the CAVE score for hemorrhagic stroke attains an AUC of 0.81, each enabling clinicians to stratify patients at the highest risk of late seizures and post-stroke epilepsy.^23^ In resource-limited clinical settings such as those characteristic of many Pakistani tertiary hospitals, deploying these evidence-based tools may allow cost-effective targeting of neurological follow-up and seizure surveillance to the highest-risk individuals, particularly those presenting with cortical or posterior cerebral artery involvement, as identified in the present study.

### Strengths

The strengths of this study include the consecutive enrolment of symptomatic stroke patients, a structured three-month prospective follow-up, and the provision of local data linking lesion location to seizure occurrence, which has rarely been reported from this region.

### Limitations

The study’s three-month follow-up was short, potentially missing late-onset seizures and some epilepsy cases. Possible inaccuracies in seizure detection and classification may have been influenced by the absence of routine EEG testing and differences in treatment methods. As a single-center tertiary hospital study, the findings are also subject to referral and selection bias and a case mix weighted towards more severe strokes, which, together with the modest sample size, may partly explain the high seizure frequency and limit how widely the results apply. Stroke severity was not formally scored, and patient comorbidities were not recorded. Larger multi-center prospective studies with formal severity scoring and comorbidity data are needed to confirm these findings.

## CONCLUSION

Post-stroke seizures were found to be a frequent complication among stroke patients, with late-onset seizures being more common. These seizures were strongly associated with cortical and posterior cerebral artery involvement. Seizures occurred in more than half of the patients followed after stroke, and cortical and posterior cerebral artery involvement were the features most often linked to their occurrence.

### Authors` Contribution:

**MR:** Conceptualization, study design, data collection, data analysis, manuscript drafting, and responsible for the accuracy of the work.

**SS:** Study supervision, critical revision of the manuscript for important intellectual content.

**MA:** Data collection, data interpretation, manuscript drafting.

**AMM:** Data collection, literature review, manuscript editing.

All authors have read and approved the final version of the manuscript.
